# AKT Inhibitors: New Weapons in the Fight Against Breast Cancer?

**DOI:** 10.3389/fphar.2021.662232

**Published:** 2021-04-29

**Authors:** Federica Martorana, Gianmarco Motta, Giuliana Pavone, Lucia Motta, Stefania Stella, Silvia Rita Vitale, Livia Manzella, Paolo Vigneri

**Affiliations:** ^1^ Department of Clinical and Experimental Medicine, University of Catania, Catania, Italy; ^2^ Center of Experimental Oncology and Hematology, A.O.U. Policlinico “G. Rodolico—S. Marco”, Catania, Italy; ^3^ Medical Oncology, A. O. U. Policlinico “G. Rodolico—S. Marco”, Catania, Italy

**Keywords:** breast cancer, AKT inhibitors, targeted therapy, PI3K/AKT/mTOR pathway, clinical trials

## Abstract

The serine/threonine kinase AKT is a key component of the PI3K/AKT/mTOR signaling pathway as it exerts a pivotal role in cell growth, proliferation, survival, and metabolism. Deregulation of this pathway is a common event in breast cancer including hormone receptor-positive (HR+) disease, HER2-amplified, and triple negative tumors. Hence, targeting AKT represents an attractive treatment option for many breast cancer subtypes, especially those resistant to conventional treatments. Several AKT inhibitors have been recently developed and two ATP-competitive compounds, capivasertib and ipatasertib, have been extensively tested in phase I and II clinical trials either alone, with chemotherapy, or with hormonal agents. Additionally, phase III trials of capivasertib and ipatasertib are already under way in HR+ and triple-negative breast cancer. While the identification of predictive biomarkers of response and resistance to AKT inhibition represents an unmet need, new combination strategies are under investigation aiming to boost the therapeutic efficacy of these drugs. As such, trials combining capivasertib and ipatasertib with CDK4/6 inhibitors, immune checkpoint inhibitors, and PARP inhibitors are currently ongoing. This review summarizes the available evidence on AKT inhibition in breast cancer, reporting both efficacy and toxicity data from clinical trials along with the available translational correlates and then focusing on the potential use of these drugs in new combination strategies.

## Introduction

**FIGURE 1:**
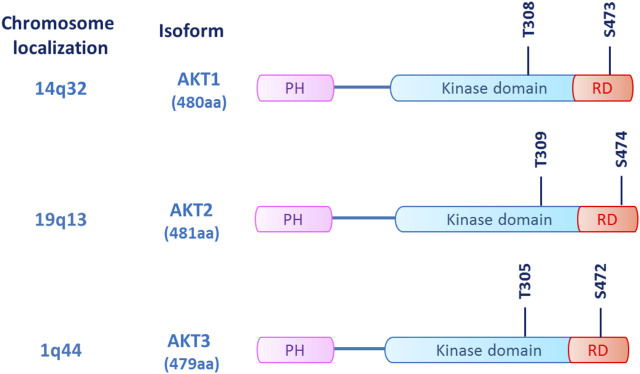
AKT structure. The three AKT isoforms (AKT1/2/3) are kinases sharing a common structure, which consists of an N-terminus pleckstrin homology (PH) domain, a large central kinase domain and a C-terminus regulatory domain (RD). The main phosphorylation sites of AKT are threonine and serine residues located in the kinase domain (T305/T308/T309) and in the regulatory domain (S472/473/474), respectively.

The serine/threonine kinase AKT, also known as protein kinase B (PKB), is a key component of the phosphatidyl-inositole-3 kinase (PI3K) intracellular pathway that exerts a pivotal role in regulating cell proliferation, survival, and metabolism (Manning and Cantley, 2007; Manning and Toker, 2017). Three AKT isoforms (*AKT1*, *AKT2*, and *AKT3*) are encoded by different genes with high sequence homology and display a conserved protein structure (Figure 1) (Matheny and Adamo, 2009). While *AKT1* and *AKT2* present a ubiquitous distribution, *AKT3* is prevalently expressed in neural cells (Hinz and Jücker, 2019). Enhanced activation of all the isoforms can be implicated in tumor development and progression, as shown in breast, ovarian, pancreatic, and prostate cancers among others (Song et al., 2019). In cancer cells, AKT1 is involved in proliferation and growth, promoting tumor initiation and suppressing apoptosis, whereas AKT2 regulates cytoskeleton dynamics, favoring invasiveness and metastatization. The role of AKT3 hyperactivation in cancer is still controversial, although a possible stimulation of cell proliferation has been hypothesized (Hinz and Jücker, 2019; Pascual and Turner, 2019).

**FIGURE 2:**
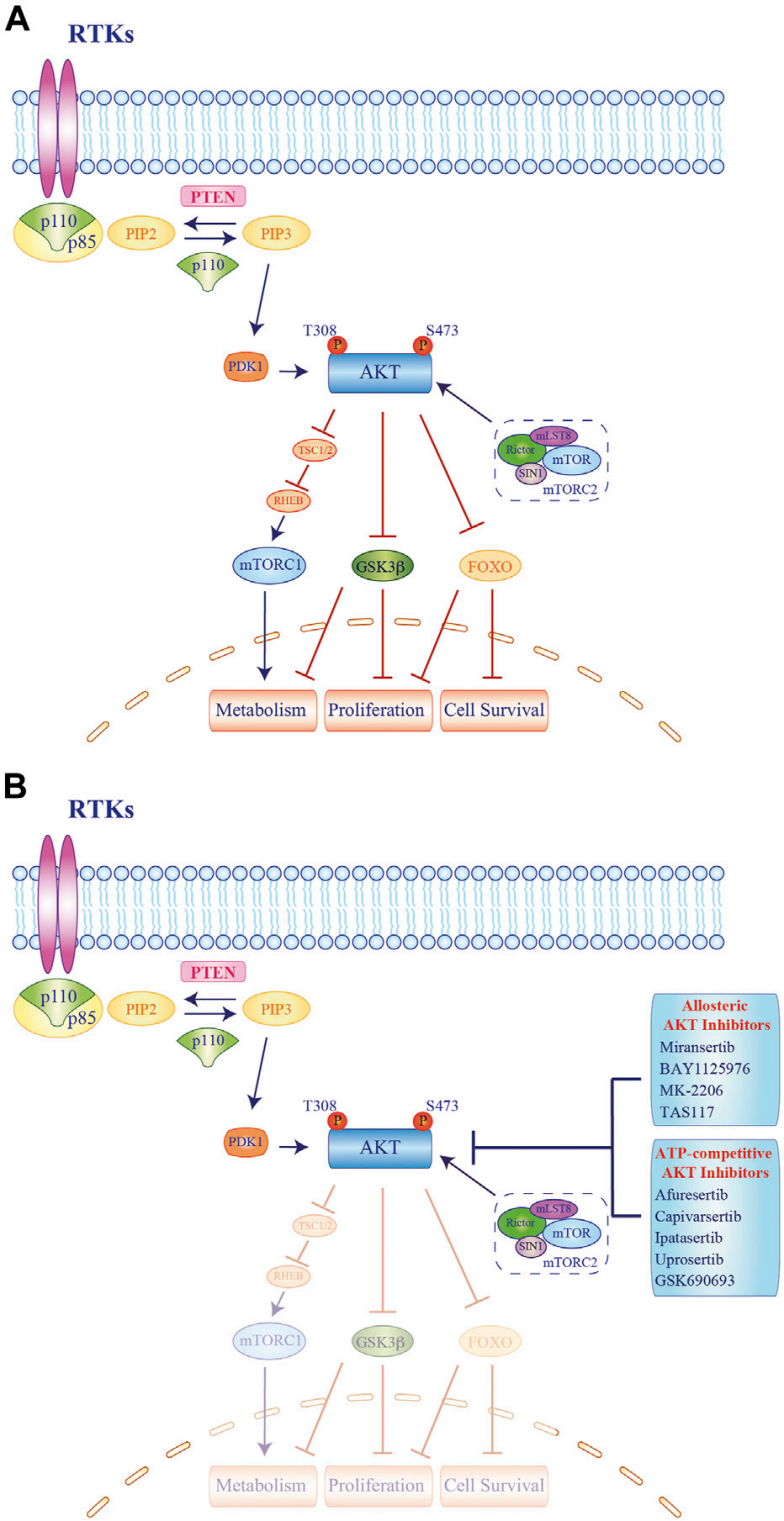
Molecular mechanisms of AKT activation and signaling cascade and schematic representation of current experimental drug combinations employing AKT inhibitors. **(A)**. Stimulation of growth factor receptor tyrosine kinases (RTKs) leads to activation of class IA phosphatidyl-inositol-3 kinase (PI3Ks). Activated class IA PI3Ks catalyze the conversion of phosphatidyl-inositol-4,5-bisphosphate (PIP2) to the second messenger phosphatidyl-inositol 3,4,5-trisphosphate (PIP3), in a reaction that can be reversed by the PIP3 phosphatase and tensin homolog deleted on chromosome 10 (PTEN). AKT and phosphoinositide-dependent kinase (PDK) 1 bind PIP3 at the plasma membrane. AKT activated by phosphorylation of the T308 residue inhibits the TSC1/2 complex, resulting in RHEB-GTP accumulation, which in turn activates mTORC1. Maximal AKT activation requires phosphorylation by mTORC2. Moreover, AKT inhibits effector proteins via phosphorylation, including glycogen synthase kinase-3 (GSK3) β and forkhead family of transcription factors (FOXO). The signaling results in the regulation of cell proliferation, survival, and metabolism. Blue arrows represent signaling activation while red bars indicate inhibitory signals. **(B)**. Activation of AKT can be inhibited by two different direct classes (Allosteric or ATP-competitive) of AKT inhibitors. Blue arrows represent signaling activation while blue bars depict inhibition of target signals. RTK = receptor tyrosine kinase.

In intracellular signaling, AKT recruitment primarily relies on the generation of phosphatidyl-inositol-triphosphate (PIP3) by PI3K, which is activated by receptor-coupled tyrosine kinases (RTKs), RAS-related GTPases, and heterotrimeric G proteins. The interaction between the PI3K regulatory subunit (p85) and the upstream effectors trough the SRC-homology 2 (SH2) domain determines the release and activation of the p110 catalytic subunit, which converts phosphatidyl-inositol (Hinz and Jücker, 2019; Song et al., 2019)-bisphosphate (PIP2) into PIP3. PIP3 recruits its substrates, including AKT and phosphoinositide-dependent kinase 1 (PDK1), to the plasma membrane. Here, AKT undergoes a double phosphorylation, one on the kinase domain (T308, T309, and T305 for AKT1, 2, and 3, respectively) by PDK1 and another on the regulatory domain (S473, S474, and S472 for AKT1, 2, and 3, respectively) by the mToR complex 2 (mTORC2), resulting in its full activation (Mundi et al., 2016). Once activated, AKT phosphorylates its downstream targets, including tuberos sclerosis complex 2 (TSC2), glycogen synthase kinase-3β (GSK3β), and the forkhead kinase transcription factors (FOXO), eventually promoting cell proliferation, metabolism, and survival (Manning and Toker, 2017; Hoxhaj and Manning, 2020). The whole pathway is negatively modulated by phosphatases, such as the phosphatase and tensin homolog (PTEN) that depho“G. Rodolico—S. Marcosphorylates PIP3 to phosphatidyl-inositol-diphosphate (PIP2), suppressing pathway activation (Figure 2A) (Lee et al., 2018).

PI3K signaling is frequently altered in breast cancer (BC) as mutations of the PI3K catalytic alpha subunit (*PIK3CA*) are common events, occurring in 9–45% of BC according to the subtype, followed by *PTEN* loss of function (13–35%) and, less frequently, *AKT* substitutions (2–4%) or amplification (5–10%) (Cancer Genome Atlas Network 2012; Guerrero-Zotano et al., 2016; Brown and Banerji, 2017; Fruman et al., 2017).

Indeed, up to 50% of hormone receptor-positive (HR+) BC and about 25% of triple-negative BC (TNBC) present PI3K/AKT pathway hyperactivation, mainly sustained by *PIK3CA* point mutations in HR + tumors and by *PTEN* loss in TNBC (Stemke-Hale et al., 2008; Cancer Genome Atlas Network 2012; Pascual and Turner, 2019; Vasan et al., 2019). This pathway is also deregulated in human epidermal growth factor receptor 2 (HER2)-enriched BC where it is involved in the development of resistance toward anti-HER2 agents, largely due to *PIK3CA* mutations (Nagata et al., 2004; Berns et al., 2007; Chandarlapaty et al., 2012). Given this biological background, targeting the key components of the PI3K/AKT pathway seems a reasonable option for the treatment of all BC subtypes. However, while mTOR and PI3K inhibitors are already approved for the treatment of advanced HR + BC patients (Baselga et al., 2012; André et al., 2021), AKT represents a novel pharmacological target.

To date, several allosteric and ATP-competitive AKT inhibitors have been synthetized and tested in clinical trials (Landel et al., 2020; Guo et al., 2019) (Figure 2B; Table 1). Allosteric inhibitors (ARQ092/miransertib; BAY1125976; MK-2206, TAS-117) were employed in advanced solid tumors in many early phase trials, but only MK-2206 has been further investigated in different BC subtypes (Biondo et al., 2011; Doi et al., 2015; Hyman et al., 2018; Schneeweiss et al., 2019; Yunokawa et al., 2019). Among ATP-competitive inhibitors, capivasertib and ipatasertib showed a favorable safety profile along with signs of activity in phase I monotherapy trials in unselected tumor types (Hyman et al., 2017; Saura et al., 2017; Banerji et al., 2018). The two compounds moved to further development and they have been extensively tested in BC patients in combination with endocrine therapy, chemotherapy, or anti-HER2 agents. While the results from some of these phase Ib/II trials are already available (Table 2), many studies are still ongoing, including phase III trials and studies exploring innovative combinations (Table 3).

**TABLE 1:** Selected AKT inhibitors and their main characteristics.

	Drug name	Compound name	Inhibited isoforms			Development phase
			1	2	3	
Allosteric	Miransertib	ARQ092	X	X	X	Phase I
	NA	BAY1125976	X	X		Phase I
	NA	MK-2206	X	X	X	Phase II
	NA	TAS-117	X	X	X	Phase II
ATP-competitive	Afuresertib	GSK2110183	X	X	X	Phase II
	Capivasertib	AZD5363	X	X	X	Phase III
	Ipatasertib	GDC0068	X	X	X	Phase III
	Uprosertib	GSK2141795	X	X	X	Phase II
	NA	GSK690693	X	X	X	Phase I—terminated

**TABLE 2:** Published trials of AKT inhibitors in breast cancer.

AKT inhibitor	Trial name	Phase	Study treatment	Study population (n. Enrolled)	Study design	Primary end point	Efficacy outcome	Ref
**CAPIVASERTIB**	STAKT	0 (WoO)	Capivasertib or placebo	Early ER + BC (neoadjuvant) (n. 48)	Randomized, double-blind	Changes in AKT pathway markers	NA	Robertson et al. (2020)
	D3610C00001	I	Capivasertib monotherapy	*PIK3CA*-mut ER + mBC (part Cb) (n. 31)	Multipart, open label	Safety	Tumor shrinkage: 46%	Banerji et al. (2018)
							ORR: 4%	
	D3610C00001	I	Capivasertib +/- Fulvestrant	*AKT1* ^E17K^ mut ER + mBC (part D) (n. 63)	Multipart, open label	Safety	ORR (monotherapy): 20% ORR (combination prior fulv.): 36%	Smyth et al. (2020)
							ORR (combination fulv. Naïve): 20%	
	FAKTION	Ib/II	Capivasertib or placebo + fulvestrant	ER + HER2- mBC, postmenopausal (n. 140)	Randomized, double-blind	PFS	mPFS: 10.3 (capiv) vs. 4.8 (pbo) months	Jones et al. (2020)
	BEECH	Ib/II	Capivasertib or placebo + Paclitaxel	ER + HER2- mBC (n. 110)	Randomized, double-blind	PFS in ITT and *PIK3CA*-mut pop	mPFS ITT: 10.9 (capiv) vs. 8.4 (pbo) months	Turner et al. (2019)
							mPFS *PIK3CA*-mut: 10.9 (capiv) vs. 10.8 (pbo) months	
	PAKT	II	Capivasertib or placebo + paclitaxel	mTNBC (n. 140)	Randomized, double-blind	PFS	mPFS: 5.9 (capiv) vs. 4.2 (pbo) months	Schmid et al. (2020a)
IPATASERTIB	FAIRLANE	II	Ipatasertib or placebo + paclitaxel	Early TNBC (neoadjuvant) (n. 151)	Randomized, double-blind	pCR in ITT and PTEN-low popul	pCR ITT: 17% (ipat) vs. 13% (pbo)	Oliveira et al. (2019)
							pCR *PTEN*-low: 16% (ipat) vs. 13% (pbo)	
	LOTUS	II	Ipatasertib or placebo + paclitaxel	mTNBC (n. 124)	Randomized, double-blind	PFS in ITT and PTEN-low popul	mPFS ITT: 6.2 (ipat) vs. 4.9 (pbo) months	Kim et al. (2017)
							mPFS PTEN-low: 6.2 (ipat) vs. 3.7 (pbo) months	
MK-2206	NA	0 (WoO)	MK-2206 monotherapy	Early BC (neoadjuvant) (n. 12)	Open label, single arm	pAKT reduction in tumor tissue	NA	Kalinsky et al. (2018)
	SU2C	Ib	MK-2206 + paclitaxel	mBC (expansion cohort) (n. 13)	Open label dose finding	MTD	ORR: 23%	Gonzalez-Angulo et al. (2015)
							CBR: 46%	
	NA	I	MK-2206 + anastrozole and/or fulvestrant	ER + HER2- mBC (n. 31)	Open label dose finding	RP2D	CBR: 36.7%	Ma et al. (2016)
	NA	I	MK-2206 + trastuzumab	HER2+ mBC^a^ (n. 27)	Open label dose finding	MTD/RP2D	ORR: 7.4%	Hudis et al. (2013)
							CBR: 22%	
	NA	I	MK-2206 +/- Lapatinib	HER2+ mBC (escalation + expansion cohort) (n. 8)	Open label dose finding	MTD/RP2D	ORR: 0%	Wisinski et al. (2016)
	NA	Ib	MK-2206 + paclitaxel + trastuzumab	HER2+ mBC (n. 12)	Open label dose finding	RP2D	ORR: 75%	Chien et al. (2016)
	NA	II	MK-2206 monotherapy	*PIK3CA*/*AKT* mut or *PTEN* altered mBC (n. 27)	Open label	ORR	ORR *PIK3CA/AKT* mut: 5.6%	Xing et al. (2019)
					Single arm		ORR *PTEN* altered: 0%	
	NA	II	MK-2206 + anastrozole	*PIK3CA*-mut ER + HER2- early BC (n. 16)	Open label	pCR	pCR rate: 0%	Ma et al. (2017)
					Single arm			
	I-SPY2	II	MK-2206 + standard NAT or standard NAT	Early BC (neoadjuvant) (n. 352)	Open label randomized adaptive	pCR	pCR e-rate overall: 35% (exp) vs. 21% (contr) pCR e-rate (ER+/HER2-): 17% (exp) vs. 13% (contr)	Chien et al. (2020)
							pCR e-rate (ER-/HER2+): 62% (exp) vs. 35% (contr)	

a These trials also enrolled patients with HER2+ advanced gastric cancer. However, only results about BC patients are reported.

Legend: AC, doxorubicin and cyclophosphamide; BC, breast cancer; Capiv, capivasertib; CBR, clinical benefit rate; Contr, control arm; ER, estrogen receptor; E-rate, estimated-rate; Exp, experimental arm; Fulv, fulvestrant; HR, hazard ratio; HT, hormone therapy; Ipat, ipatasertib; ITT, intention-to-treat; m, metastatic; mPFS, median progression-free survival MTD, maximum tolerated dose; Mut, mutated; NA, not applicable; NAT, neoadijuvant therapy; ORR, objective response rate; Pbo, placebo; pCR, pathologic complete response, Popul: population; RP2D, recommended phase II dose; TNBC, triple-negative breast cancer; WoO, window of opportunity.

**TABLE 3:** Ongoing trials of AKT inhibitors in breast cancer.

AKT inhibitor	Trial identifier (name)	Phase	Study treatment	Study population	Study design
CAPIVASERTIB	NCT03310541^a^	I	Capivasertib + Fulvestrant	HR + mBC *AKT* mut after fulvestrant	Multi-cohort, nonrandomized
	NCT02338622 (ComPAKT)	I	Capivasertib + Olaparib	Advanced solid tumors	Multi-cohort, nonrandomized
	NCT03772561 (MEDIPAC)	I	Capivasertib + Olaparib + Durvalumab	Advanced solid tumors	Single arm
	NCT03742102 (BEGONIA)^a^	I/II	Capivasertib + Paclitaxel + Durvalumab	mTNBC	Multi-cohort, randomized
	NCT02576444 (OLAPCO)^a^	II	Capivasertib + Olaparib	Advanced solid tumors with PI3K/AKT pathway alterations	Multi-cohort, nonrandomized
	NCT03997123 (CAPItello290)	III	Capivasertib + Paclitaxel vs. Pbo + Paclitaxel	1L mTNBC	Randomized, double-blind
	NCT04305496 (CAPItello-291)	III	Capivasertib + Fulvestrant vs. Pbo + Fulvestrant	HR+/HER2- mBC, after an AI	Randomized, double-blind
IPATASERTIB	NCT03959891 (TAKTIC)	I	Ipatasertib + AI or Ipatasertib + Fulvestrant or Ipatasertib + Fulvestrant + Palbociclib	HR+/HER2- mBC, after CDK4/6-i	Multi-cohort, nonrandomized
	NCT04253561 (IPATHER)	Ib	Ipatasertib + Trastuzumab + Pertuzumab	HER2+ mBC *PI3KCA*-mut (1L mantainance)	Single arm
	NCT03800836	Ib	Ipatasertib + Atezolizumab + Paclitaxel or Ipatasertib + Atezolizumab + Nab-Paclitaxel or Ipatasertib + Atezolizumab + AC → Paclitaxel	1L mTNBC	Multi-cohort, nonrandomized
	NCT03280563 (MORPHEUS HR + BC)^a^	Ib/II	Ipatasertib + Atezolizumab or Fulvestrant	HR+/HER2- mBC, after 1/2L CDK4/6-i	Multi-cohort, randomized
	NCT03424005 (MORPHEUS TNBC)^a^	Ib/II	Ipatasertib + Atezolizumab or Capecitabine	mTNBC	Multi-cohort, randomized
	NCT03840200	Ib/II	Ipatasertib + Rucaparib	HER2- mBC, EOC or PC	Single arm
	NCT03853707	I/II	Ipatasertib + Carboplatin + Paclitaxel or Ipatasertib + Carboplatin or Ipatasertib + Capecitabine + Atezolizumab	mTNBC	Multi-cohort, nonrandomized
	NCT03673787 (Ice-CAP)^a^	I/II	Ipatasertib + Atezolizumab	Advanced solid tumors with PI3K/AKT pathway alterations	Multi-cohort, nonrandomized
	NCT04434040	II	Ipatasertib + Atezolizumab	eTNBC with ctDNA after surgery	Single arm
	NCT04464174 (PATHFINDER)	IIa	Ipatasertib + Capecitabine or Ipatasertib + Eribulin or Ipatasertib + Carboplatin + Gemcitabine	mTNBC	Multi-cohort, nonrandomized
	NCT03395899 (ECLIPSE)^a^	II	Ipatasertib + Atezolizumab or Ipatasertib + Bevacizumab + Atezolizumab or Atezolizumab	HR+/HER2- eBC	Multi-cohort, randomized
	NCT03337724 (IPATunity130)	III	Ipatasertib + Paclitaxel or Pbo + Paclitaxel	mTNBC (cohort A) or HR+/HER2- mBC (cohort B)	Randomized, double-blind
	NCT04650581 (FINER)	III	Ipatasertib + Fulvestrant or Pbo + Fulvestrant	HR+/HER2- mBC after 1L AI + CDK4/6-i	Randomized, double-blind
	NCT04060862 (IPATunity150)	III	Ipatasertib + Palbociclib + Fulvestrant or Pbo + Palbociclib + Fulvestrant	HR+/HER2- mBC	Randomized, double-blind
	NCT04177108 (IPATunity170)	III	Ipatasertib + Paclitaxel + Atezolizumab	1L mTNBC	Randomized, double-blind

a Trials with multiple experimental arms. The table shows only the experimental arms containing AKT inhibitors and the arm with the active comparator, when applicable.

Legend: 1/2L, first or second line; AC, doxorubicine and cyclophosphamide; AI, aromatase inhibitor; BC, breast cancer; CDK4/6-i CDK4/6 inhibitor; ctDNA, circulating tumor DNA; CeBC, early breast cancer; EOC, epithelial ovarian cancer; HR + , hormone receptor positive; mBC, metastatic breast cancer; mut, mutated; Pbo, placebo; PC, prostate cancer; TNBC, triple-negative breast cancer.

Here, we review the existing clinical evidence on AKT inhibitors in BC and we also report the available translational correlations. We then focus on the toxicity spectrum of these drugs and finally discuss novel combination strategies, which are under investigation.

## Clinical Trials of AKT Inhibitors in BC Subtypes

### AKT Inhibitors in HR+/HER-BC

Hyperactivation of the PI3K/AKT/mTOR pathway represents an oncogenic driver and can determine resistance toward endocrine therapies in HR + BC (Massarweh and Schiff, 2007). Since AKT is altered in about 7% of HR + BC, it represents a potential therapeutic target which can be exploited in combination strategies with endocrine therapy or chemotherapy (Miller et al., 2011).

AKT inhibitors and anti-estrogen agents showed preliminary signs of activity in early trials conducted in HR + metastatic breast cancer. In a phase I study enrolling heavily pretreated patients with HR+, AKT^E17K^-mutated BC, the combination of capivasertib and fulvestrant determined objective responses in both fulvestrant-naïve (*n* = 15) and fulvestrant-pretreated (*n* = 28) women (objective response rate [ORR] 20 and 36%, respectively) (Smyth et al., 2020). Similar results were observed in a phase I trial evaluating the allosteric inhibitor MK-2206 in combination with fulvestrant, anastrozole, or both in ER+, HER2-metastatic BC patients (*n* = 31). ORR in the overall population was 15.4% with no correlation between PIK3CA mutational status and responses (Ma et al., 2016).

The combination of capivasertib and fulvestrant was further explored in a randomized, placebo-controlled phase II trial (FAKTION), which enrolled postmenopausal women with HR+/HER2-advanced BC relapsing after or progressing on an aromatase inhibitor (AI). Progression-free survival (PFS) was the primary endpoint. Of the 140 enrolled patients, 69 received capivasertib plus fulvestrant and 71 received placebo plus fulvestrant. In the overall population, the addition of the AKT inhibitor to endocrine therapy provided a statistically significant 5.5 months gain in median PFS (10.3 months in the experimental arm vs. 4.8 months in the control arm [HR 0.58; 95% CI: 0.39–0.84, p 0.004]). However, the same magnitude of benefit was not observed in the PI3K/PTEN-altered tumors, defined per protocol as exon 9 or 20 PIK3CA-mutated or PTEN null by immunohistochemistry (IHC). In this subset of patients (*n* = 59), median PFS was 9.5 months in the capivasertib plus fulvestrant group and 5.2 months in the placebo plus fulvestrant group [HR 0.59; CI: 0.34–1.03; p 0.064] (Jones et al., 2020). Despite overall survival (OS) not being mature at the time of data cutoff, a trend in favor of the experimental arm emerged. A large randomized confirmatory phase III trial (CAPItello-291) of capivasertib and fulvestrant is currently ongoing in HR+/HER-metastatic BC patients after the failure of an AI-based therapy. Trial population will be stratified according to the PI3K/AKT/PTEN mutational status in order to clarify the possible role of these biomarkers (Turner et al., 2020b). Another phase III randomized, placebo-controlled trial of ipatasertib and fulvestrant is ongoing and will evaluate a metastatic HR+/HER-BC population progressing after first-line therapy with an AI and a cyclin-dependent kinase 4/6 inhibitor (CDK4/6) (FINER, NCT04650581).

The use of AKT inhibitors and endocrine therapy in early stage HR + BC are still scarce. In a phase II trial, MK-2206 and anastrozole were administered in the neoadjuvant setting to women with stage II-III, HR+/HER2-PIK3CA mutant BC. After the enrollment of the first 16 patients, no pathological complete responses were observed and the study was closed to accrual for futility (Ma et al., 2017).

Chemotherapeutic agents can also be combined with AKT inhibitors for the treatment of HR + BC. In the phase Ib/II BEECH trial capivasertib was administered along with weekly paclitaxel to HR + metastatic BC patients. The study included a nonrandomized safety run-in part for the identification of the RP2D (recommended phase 2 dose) and a subsequent randomized placebo-controlled phase II part, in which patients were stratified according to PIK3CA mutational status. Primary endpoint of the phase II part was PFS in the overall population and in the PIK3CA-mutated subgroup. In the overall population, a not statistically significant difference in median PFS emerged with the addition of capivasertib to weekly paclitaxel (*n* = 110) (10.9 months in experimental arm [*n* = 54] vs. 8.4 months in the control arm [*n* = 56] [HR 0.80; p 0.308]). Median PFS was superimposable between the two treatment arms in the PIK3CA-mutated population (*n* = 51) (10.9 months with capivasertib plus paclitaxel [*n* = 26] vs. 10.8 months with placebo plus paclitaxel [*n* = 25][HR 1.11; p 0.760]) (Turner et al., 2019). An ongoing phase III multi-cohort trial (IPATunity130) randomizes patients with PI3K/AKT1/PTEN altered metastatic BC to receive paclitaxel plus ipatasertib or placebo as first chemotherapy line. Results about cohort B, enrolling HR+/HER2 patients not eligible for endocrine therapy have been recently reported. Two-hundred twenty-two patients were included in this cohort, and randomized 2:1. Median PFS, the primary endpoint, was identical in the two arms (9.3 months; HR 1; CI 0.71–1.4), while OS data were still immature (Turner et al., 2020a).

In the neoadjuvant setting, MK-2206 was tested with standard preoperative therapy in a cohort of the platform adaptive randomized phase II I-SPY2 trial. However, the estimated probability to achieve a pathological complete response (pCR) was not significantly different between the experimental and control arm (17% vs. 13%) in the HR+/HER2-subset of patients (Chien et al., 2020).

### AKT Inhibitors in TNBC

The PI3K/AKT signaling pathway is frequently hyperactivated in TNBC due to *PIK3CA* or *AKT1* mutations and/or *PTEN* inactivation (Cancer Genome Atlas Network 2012; Millis et al., 2015; LoRusso, 2016; Nik-Zainal et al., 2016). According to preclinical evidence, AKT inhibition can increase chemosensitivity in TNBC, eventually overcoming chemoresistance in this disease subset (Davies et al., 2012; Yan et al., 2013; Isakoff et al., 2020). Hence, several trials have investigated AKT inhibitors in association with chemotherapy for TNBC.

Two randomized placebo-controlled phase II trials (PAKT and LOTUS) evaluated the combination of an ATP-competitive inhibitor (i.e., capivasertib and ipatasertib) with weekly paclitaxel for the first-line treatment of advanced TNBC (Kim et al., 2017; Schmid et al., 2020a). The PAKT trial randomized 140 patients to receive capivasertib plus paclitaxel (*n* = 70) or placebo plus paclitaxel (*n* = 70). The population was stratified according to the *PIK3CA/AKT1/PTEN* mutational status assessed with next generation sequencing (NGS). The primary endpoint was median PFS in the intention-to-treat population and it was numerically longer in the experimental arm (5.9 months) compared to the control arm (4.2 months) (HR 0.74; 95% CI: 0.5–1.08, one-sided p = 0.06). However, progression-free survival was significantly extended with capivasertib in the *PIK3CA/AKT1/PTEN* mutated subpopulation (9.3 months vs. 3.7 months; HR 0.3; 95% CI: 0.11–0.79; p 0.1) (Schmid et al., 2020a). Updated results after 40 months of follow-up showed a favorable trend in terms of OS for capivasertib plus paclitaxel, regardless of the *PIK3CA/AKT/PTEN* mutational status (median OS in the overall population 19.1 months vs. 13.5 months; HR 0.7; 95% CI: 0.47–1.05; p = 0.085) (Schmid et al., 2021a). To further elucidate the efficacy of this regimen, a phase III randomized, placebo-controlled trial of capivasertib and paclitaxel for the first-line treatment of advanced TNBC (CAPItello290) opened for accrual in May 2020 (Schmid et al., 2020b).

The results of the PAKT trial are consistent with those of the LOTUS trial, which compared ipatasertib plus paclitaxel with placebo plus paclitaxel in 124 TNBC patients, previously untreated for advanced disease. The trial had two co-primary endpoints: PFS in both the overall and in the PTEN-low (by immunohistochemistry; *n* = 48) population. Median PFS was longer in the experimental arm, both in the intention-to-treat (6.2 months vs. 4.9 months; HR 0.6; 95% CI: 0.37–0.98; p = 0.03) and in the PTEN-low population (6.2 months vs. 3.7 months; HR 0.59; 95% CI: 0.26–1.32; p = 0.18). The benefit of ipatasertib was more pronounced in the *PIK3CA/AKT/PTEN*-altered population (*n* = 42), identified by NGS. In this group, median PFS was 9.0 months in the experimental arm compared with 4.9 months in the control arm (HR 0·44; 95% CI: 0.20–0.99, p = 0.04) (Kim et al., 2017). The final analysis, conducted after an extended follow-up, showed a nonstatistically significant prolonged OS in the ipatasertib plus paclitaxel group compared to the placebo plus paclitaxel group (25.8 months vs. 16.9 months; HR 0.8; 95% CI: 0.5–1.28). A numerical survival benefit was observed, regardless of PTEN expression and *PIK3CA/AKT/PTEN* mutational status (Dent et al., 2020b). The combination of ipatasertib and paclitaxel is under further investigation in cohort A of the confirmatory phase III randomized IPATunity130 trial, which enrolls advanced, previously untreated, *PIK3CA/AKT/PTEN*-altered TNBC patients. Data from the primary analysis failed to demonstrate any significant difference in terms of PFS between the two treatment arms (7.4 months vs. 6.1 months; HR 1.02; p 0.9), while OS results were still immature (Dent et al., 2020a). Additionally, the combination of ipatasertib with a non-taxane–based chemotherapy in mTNBC patients is currently under evaluation in the phase II PATHFINDER trial.

In the early-stage setting, a phase II randomized trial evaluated the use of AKT inhibitors in TNBC. The FAIRLANE trial compared ipatasertib and paclitaxel with placebo plus paclitaxel as a 12-week neoadjuvant treatment for TNBC (cT ≥ 1.5 cm; cN 0–2) patients. Co-primary endpoints were pCR in the overall and PTEN-low population, defined by IHC. One-hundred fifty-one patients were randomized in 1:1 ratio and 35 of them had a PTEN-low tumor. In the overall population, pCR was achieved in 17% of patients in the experimental arm and in 13% of patients in the control arm, with comparable rates observed in the PTEN-low population (pCR 16% vs. 13%). Response rates assessed with MRI, a secondary endpoint, were numerically superior in the ipatasertib arm, especially among PTEN-low tumors (Oliveira et al., 2019).

### AKT Inhibitors in HER2+ BC

The PI3K pathway is altered in up to 50% of HER2-enriched BC, mainly as a consequence of PIK3CA mutations or PTEN loss (Nagata et al., 2004; Isakoff et al., 2005; Wisinski et al., 2016). Hyperactivation of this pathway contributes to the development of primary and acquired resistance toward HER2-targeted therapies (Nagata et al., 2004; Berns et al., 2007; Chandarlapaty et al., 2012; Loibl et al., 2014; Guerrero-Zotano et al., 2016; Fujimoto et al., 2020). Hence, targeting AKT in HER2-positive BC has a biological rationale which is currently under evaluation in several clinical trials.

Early phase studies tested MK-2206 along with trastuzumab or lapatinib in HER2-enriched tumors, including BC. The combination of MK-2206 and trastuzumab showed preliminary signals of efficacy in a phase I trial conducted in HER2-positive advanced breast and gastroesophageal tumors. Among the 27 BC patients enrolled, all pretreated with trastuzumab, one complete response and one partial response occurred (Hudis et al., 2013). Another phase Ib trial tested MK-2206 with trastuzumab and weekly paclitaxel, enrolling patients with HER2+ solid tumors, including 12 BC patients. Three complete responses and six partial responses were observed in this selected population, with a remarkable 75% ORR (Chien et al., 2016). Conversely, no objective responses were registered in the phase I trial of MK-2206 and lapatinib, which enrolled eight HER2-positive BC patients. However, two patients experienced disease stabilization for more than 6 months (Wisinski et al., 2016).

The addition of ipatasertib to standard first-line maintenance with trastuzumab and pertuzumab (after a taxane-based chemotherapy) is under investigation in a single arm phase Ib trial (SOLTI-1507/IPATHER) in patients with HER2+/PIK3CA-mutated advanced BC. Beside the identification of the maximum tolerated dose of ipatasertib (primary endpoint), this trial will provide preliminary data about the potential efficacy of this regimen (Oliveira et al., 2020).

The randomized phase II I-SPY2 platform trial tested the combination of MK-2206 and standard preoperative therapy in stage II-III BC patients. Overall, 44 patients presented a HER2-positive disease. 34 of them were assigned to the experimental arm, while 10 were treated in the control arm. According to the adaptive Bayesian study design, the posterior probability to obtain a pCR was significantly increased by the addition of MK-2206 to standard therapy (48% in the experimental arm vs. 29% in the control arm), especially in the HR-negative/HER2-positive population (62% in the experimental arm vs. 35% in the control arm) (Chien et al., 2020). Despite these encouraging results, the combination has not been further investigated.

## Biomarkers of Response to AKT Inhibitors

The identification of biomarkers of response to AKT inhibitors is of pivotal importance to maximize the potential efficacy of these targeted agents, pursuing a “personalized medicine” approach for breast cancer patients. To this end, correlatives and translational studies have been extensively conducted in the context of clinical trials, but results are still inconclusive.

Determining the phosphorylation level of downstream effectors is useful to establish whether AKT inhibition effectively downregulates PI3K hyperactivation. In the STAKT trial, patients with newly diagnosed HR-positive early BC received capivasertib for 4.5 days prior to surgery. Compared with baseline levels, posttreatment phosphorylation of the downstream effectors GSK3β, PRAS40, and S6 was significantly decreased, indicating that capivasertib effectively blocked its target (Robertson et al., 2020). A meaningful decrease of phospho-GSK3β was also observed among metastatic BC patients treated with capivasertib in phase I/II trials (Banerji et al., 2018; Turner et al., 2019). Conversely, only a modest reduction of pS6, PTEN, and stathmin phosphorylation emerged in a window of opportunity (WoO) trial evaluating MK-2206 in early BC patients, irrespectively of their intrinsic subtype (Kalinsky et al., 2018). A more comprehensive phospho-proteomic analysis was carried out on the early HR-negative/HER2-positive BC and TNBC population of the I-SPY2 trial, with high pAKT, pSGK, pmTOR, and pTSC2 levels prior to neoadjuvant treatment with MK-2206 and standard therapy positively correlating with pCR rates (Wolf et al., 2020).

The Ki-67 proliferation index has also been evaluated in neoadjuvant trials in order to assess AKT inhibitors’ efficacy. In line with the reported data mentioned above, a decrease in Ki-67 was observed after treatment with capivasertib in the STAKT trial, while no significant differences in pre- and posttreatment Ki-67 emerged in the MK-2206 WoO trial (Kalinsky et al., 2018; Robertson et al., 2020).

Since preclinical evidences suggest that alterations in the PI3K/AKT pathway may confer sensitivity to AKT inhibition in BC models (Davies et al., 2012; Sangai et al., 2012; Lin et al., 2013), *PIK3CA*, *AKT*, and *PTEN* mutations along with PTEN expression levels have been extensively investigated in clinical trials testing AKT inhibitors. Mutational status of selected genes was assessed on tumor tissue or circulating tumor DNA (ctDNA) with different methods, including Sanger sequencing (Ma et al., 2017), real-time PCR (RT-PCR) (Turner et al., 2019), digital droplet PCR (ddPCR) (Jones et al., 2020; Smyth et al., 2020), and NGS (Kim et al., 2017; Oliveira et al., 2019; Schmid et al., 2020a), whereas PTEN expression was universally determined by tissue IHC.

Overall, a lack of correlation between PI3K/AKT pathway alterations and efficacy of AKT inhibitors consistently emerged from trials conducted in HR-positive BC patients (Ma et al., 2016; Turner et al., 2019; Jones et al., 2020). A number of explanations have been proposed for this phenomenon. Whether suboptimal drug exposure due to toxicities or a small sample size may have influenced the results of the MK-2206 phase I trial in advanced HR-positive/HER2-negative BC patients (Ma et al., 2016), the crosstalk between the estrogen receptor and PI3K signaling is the most likely cause for the lack of additional benefit from capivasertib observed in the *PI3K/AKT/PTEN* altered population of the FAKTION study (Jones et al., 2020). Additionally, insufficient inhibition of the PI3K/AKT pathway during paclitaxel treatment may have determined a superimposable outcome between *PIK3CA* mutant and *PIK3CA* wild-type patients in the BEECH trial (Turner et al., 2019). In the same trial population, a reduction in total ctDNA levels during treatment strongly correlated with PFS in both arms. However, this finding was not influenced by *PIK3CA* mutational status, strengthening the assumption that the effect of AKT inhibitors does not mirror PI3K/AKT hyperactivation in HR-positive BC (Hrebien et al., 2019).

Conversely, a trend toward better outcomes in patients with altered PI3K/AKT signaling emerged in studies enrolling TNBC patients (Kim et al., 2017; Oliveira et al., 2019; Schmid et al., 2020a). In the PAKT and LOTUS trials, combining capivasertib or ipatasertib with paclitaxel was more beneficial among patients with advanced TNBC harboring a mutation in *PIK3CA*, *AKT,* or *PTEN* detected by NGS (Kim et al., 2017; Schmid et al., 2020a). However, the same magnitude of benefit was not observed in LOTUS among PTEN-low patients. Since a greater proportion of patients presented low PTEN expression at the protein level compared with those who had a mutation or a copy number loss, it is conceivable that non-genomic mechanisms of *PTEN* disfunction are weaker in hyperactivating the PI3K/AKT pathway (Kim et al., 2017).

Data concerning PI3K pathway activation and response to AKT inhibitors in HER2-positive patients are difficult to evaluate, given the small number of patients enrolled in early phase trials (Hudis et al., 2013; Chien et al., 2016; Wisinski et al., 2016). However, according to a phospho-proteomic analysis, hyperactivation of the PI3K/AKT pathway prior to neoadjuvant treatment with MK-2206 positively correlated with pCR rates in the HER2-enriched population of the I-SPY2 trial (Wolf et al., 2020). Unexpectedly, in the same analysis, a high phosphorylation rate in proteins of the PI3K pathway was negatively associated with disease response in the TNBC group (Chien et al., 2016). While this discrepancy remains unexplained, these findings further indicate that the correlation between PI3K/AKT activation and efficacy of the AKT inhibitors depends on a complex—and as yet only partially understood—specific biological context of each BC subgroup.

Limited evidence is available on the role of immunomodulation as a biomarker of response to AKT inhibitors. In the FAIRLANE trial, an immune score was calculated among early TNBC patients receiving ipatasertib or placebo plus paclitaxel in the neoadjuvant setting. While an increase of this score during treatment significantly correlated with tumor response in the control arm, the same association was not found in the experimental group (Oliveira et al., 2019). Consistently, in the I-SPY2 trial, whole-transcriptome analysis and extensive protein arrays failed to show a correlation between the immune signature and response to preoperative MK-2206 among TNBC patients. However, this signature was significantly associated with tumor response in the HER2-enriched population, suggesting that—in this BC subtype—the immune environment may play an important role in mediating response to AKT inhibition (Wolf et al., 2020).

## Toxicity Spectrum of AKT Inhibitors

A well-known spectrum of toxicities derives from the pharmacological inhibition of the PI3K/AKT pathway (Chia et al., 2015; Esposito et al., 2019; Zhang et al., 2019). Indeed, safety represents one of the main issues for the clinical development of agents blocking AKT activity. Additionally, the close homology between the three AKT isoforms hinders the development of isoform-specific inhibitors, which may reduce their toxicity burden (Nunnery and Mayer, 2020).

Diarrhea has been reported with PI3K inhibitors (e.g., idelalisib) and is likely caused by an immune-mediated mechanism (Louie et al., 2015; Weidner et al., 2015). Even though the pathogenesis of AKT suppression-induced diarrhea is still unclear, it was the most common adverse event (AE) of any grade observed with ATP-competitive inhibitors, with a peak incidence of 93% in the experimental arm of the LOTUS trial (ipatasertib + paclitaxel) (Kim et al., 2017). Indeed, diarrhea was a frequent dose-limiting toxicity in dose-finding trials (DLT) (Banerji et al., 2018; Turner et al., 2019), besides determining several dose reductions and treatment discontinuations. However, it was mild or moderate in the majority of cases, with an incidence of grade 3 or higher (G ≥ 3) events of 8–23% and rare G4 occurrences. This AE usually had an early onset and was reversible after treatment discontinuation and proper management with antidiarrheal agents, such as loperamide (Hyman et al., 2017; Kim et al., 2017; Saura et al., 2017; Banerji et al., 2018; Oliveira et al., 2019; Turner et al., 2019; Schmid et al., 2020a; Jones et al., 2020; Smyth et al., 2020; Tolcher et al., 2020). Despite not planned in clinical trials, the prophylactic use of antidiarrheal medications may improve the tolerability of AKT inhibitors and warrants further investigation in future trials.

Dermatological toxicity represents another concern when employing AKT inhibitors. Its pathogenesis presumably relies on PI3K/AKT involvement in keratinocyte differentiation and survival (Calautti et al., 2005). Skin toxicity was observed with all AKT inhibitors, but it was particularly common with MK-2206, where it represented the most frequent DLT, reaching a G ≥ 3 rate of up to 29% (Hudis et al., 2013; Chien et al., 2016; Ma et al., 2016; Ma et al., 2017; Kalinsky et al., 2018; Xing et al., 2019; Chien et al., 2020). Skin rash also occurred in phase I-II trials of capivasertib or ipatasertib, but the incidence of G ≥ 3 events was lower with these drugs (Hudis et al., 2013; Chien et al., 2016; Ma et al., 2016; Ma et al., 2017; Kalinsky et al., 2018; Xing et al., 2019; Chien et al., 2020). In clinical studies, rash had usually a maculopapular appearance and was inconsistently associated with pruritus. It was managed with topical steroids and drug interruption with or without dose reduction, when needed. However, more severe cases required systemic steroids and led to treatment discontinuation in some cases (Hudis et al., 2013; Chien et al., 2016; Ma et al., 2016; Ma et al., 2017; Kalinsky et al., 2018; Xing et al., 2019; Chien et al., 2020). Given the frequent occurrence of rash with MK-2206, preemptive oral prednisone was administered in some trials, with no clear benefit (Ma et al., 2016; Ma et al., 2017; Kalinsky et al., 2018).

Hyperglycemia is a well-described consequence of PI3K/AKT pathway inhibition (Esposito et al., 2019; Nunnery and Mayer, 2019). It is determined by a perturbation in the insulin-mediated glucose homeostasis, which largely depends on PI3K signaling via GSK3β and FOXO (Zhang et al., 2019). The incidence of hyperglycemia of any grade in clinical trials of AKT inhibitors varies broadly, going from 92% with MK-2206 and anastrozole to 4% with ipatasertib and paclitaxel (Ma et al., 2016; Oliveira et al., 2019). Phase I trials of capivasertib and ipatasertib display a considerable incidence of G ≥ 3 hyperglycemia, without reports of ketoacidosis or hyperosmolar coma (Hyman et al., 2017; Saura et al., 2017; Banerji et al., 2018). Proper patient selection (i.e., exclusion of subjects with uncontrolled diabetes) and accurate monitoring of glucose blood levels may have played a role in the different incidence of this toxicity across the studies. As already reported for mTOR and PI3K inhibitors, dietary intervention, glucose lowering medications, and treatment interruption with or without dose reduction can be helpful when hyperglycemia occurs, although multidisciplinary management is highly recommended (Esposito et al., 2019; Nunnery and Mayer, 2019).

Additional on-target AEs (e.g., hepatic toxicity (Hudis et al., 2013; Saura et al., 2017), hypertension (Chien et al., 2016; Jones et al., 2020), hypercholesterolemia (Jones et al., 2020), and stomatitis (Schmid et al., 2020a)) already observed with inhibitors of the PI3K/AKT pathway occurred less frequently and were rarely severe (Chia et al., 2015; Esposito et al., 2019). Nausea, fatigue, and neutropenia were common, but were mainly registered in trials evaluating AKT inhibitors in combination with chemotherapeutic agents, to which they were largely attributable (Kim et al., 2017; Oliveira et al., 2019; Turner et al., 2019; Schmid et al., 2020a).

## Future Perspectives

Several strategies are under evaluation in order to improve the efficacy of AKT inhibitors with multiple new compounds currently in their early development. A hybrid covalent-allosteric AKT inhibitor (borussertib) has recently been synthetized showing preclinical activity in cell lines and xenograft models (Weisner et al., 2019), while a nanoparticle-encapsulated version of capivasertib was tested in radio-resistant models of oral cavity cancer (Lang et al., 2020). However, the most intuitive approach to increase AKT inhibitors activity in BC is to combine them with biological agents targeting different pathways favoring cancer cell survival and proliferation. To this end, many approaches are being evaluated in clinical trials.

Since *AKT* alterations may confer resistance to CDK4/6 inhibition (Wander et al., 2018), a phase Ib trial (TAKTIC) is evaluating the addition of ipatasertib to endocrine therapy (an AI or fulvestrant) with or without palbociclib in patients with HR + metastatic BC progressing on a prior CDKI. Preliminary results on the first 12 patients enrolled in cohort C (ipatasertib + fulvestrant + palbociclib) were reported at ASCO 2020. The triplet showed signals of clinical activity in this heavily pretreated population with proven resistance to CDKIs, with two partial responses and three stable disease registered (Wander et al., 2020). IPATunity150 is a phase III randomized trial with a preliminary safety run-in cohort, which will compare the combination of ipatasertib + fulvestrant + palbociclib and placebo + fulvestrant + palbociclib in a population of endocrine resistant HR + BC, naïve to CDK4/6 inhibitors. The trial is open to accrual but no data have yet been posted.

Complex molecular networks are intertwined between cancer intracellular signaling and mechanisms of tumoral immune escape, such as the programmed death-1 (PD-1)/programmed death ligand-1 (PD-L1) system (Ai et al., 2020). Hence, combining PD-1/PD-L1 inhibitors with AKT inhibitors may enhance the activity of both compounds. Indeed, a plethora of studies are investigating the association between AKT inhibitors and immunotherapeutic agents with or without chemotherapy. Encouraging results emerged from a phase Ib trial (CO40151/NCT03800836) combining ipatasertib, an anti PD-L1 (atezolizumab) and a chemotherapeutic agent (adriamicine plus cyclophosphamide, paclitaxel or nanoparticle-albumin bound paclitaxel) for the first-line treatment of metastatic TNBC. Updated results on 114 patients showed a 54% ORR (95% CI 44–63%) and a median PFS of 7.2 months (95% CI 5.5–7.4 months), irrespective of PD-L1 expression and of *PI3K/AKT/PTEN* mutational status (Schmid et al., 2019; Schmid et al., 2021b). Many studies associating AKT inhibitors with immunotherapy are currently enrolling patients. Some combine ipatasertib and atezolizumab, either in advanced solid tumors, in HR + or in triple negative metastatic breast cancer patients (Ice-CAP; MORPHEUS HR+ and MORPHEUS TNBC). Other studies are investigating the HR + neoadjuvant setting (ECLIPSE) or the TNBC adjuvant setting (NCT04434040). Additional trials incorporate an AKT inhibitor (capivasertib or ipatasertib), an anti PD-L1 (durvalumab or atezolizumab) and chemotherapeutic agents for the treatment of advanced TNBC (BEGONIA; IPATunity170; NCT03853707). Preliminary results from these trials are still unavailable.

Preclinical evidence also supports the simultaneous inhibition of poly-adenosyl-ribose-polymerase (PARP) and the PI3K/AKT pathway as this approach may confer sensitivity toward PARP inhibitors, regardless of the *BRCA* mutational status (Ibrahim et al., 2012; Juvekar et al., 2012; Rehman et al., 2012; Mo et al., 2016). To prove this principle, a phase I trial (ComPAKT) combined the PARP inhibitor olaparib with capivasertib in patients with advanced solid tumors. Expansion cohorts included subjects with *BRCA1/2* mutant and *BRCA1/2* wild type tumors, the latter with or without DNA damage repair deficiency or alterations in the PI3K/AKT pathway. Eighteen of the 64 enrolled patients had advanced BC. Eight of them (44%) experienced a clinical benefit (i.e., partial response or stable disease ≥4 months), six displaying a germline defect in homologous recombination (*BRCA1/2*, *PALB2*, or *RAD51D* mutations) and 2 a *PIK3CA* somatic mutation (Yap et al., 2020). A phase II platform trial (OLAPCO) is evaluating the combination of olaparib and capivasertib in patients with advanced solid tumors harboring *PI3K*, *AKT*, *PTEN,* or *ARID1A* mutations, while another (NCT03840200) is testing rucaparib and ipatasertib in advanced breast, ovarian, and prostate cancer. These studies are already closed to accrual and results are awaited (Eder et al., 2018). Finally, an ongoing phase I trial (MEDIPAC) is testing the triplet capivasertib, durvalumab, and olaparib.

## Discussion

Given the key role of PI3K signaling in breast cancer, the inhibition of this pathway has been pursued by several means in the last decade. While inhibitors of mTOR (everolimus) and of the PI3K catalytic alfa subunit (alpelisib) have already entered clinical practice, many compounds targeting other pathway components are in clinical development, and AKT inhibitors are among these. Of note, AKT seems to represent a transversal target across the BC intrinsic subtypes (i.e., luminal HER2-, HER2 enriched, and triple negative) and its inhibition has, therefore, been explored in all three categories.

However, targeting the AKT pathway presents several challenges both in terms of efficacy and safety, as the results of trials evaluating these compounds have shown.

The disappointing outcome of some phase II and III trials combining capivasertib or ipatasertib with endocrine therapy and chemotherapy may indicate that AKT inhibition alone is not sufficient to tackle hyperactivation of the PI3K pathway. The dismal results of neoadjuvant trials represent a proof-of-principle for this assumption. This might mean that AKT mutations do not represent driver events in cancer cells. Another potential explanation for this phenomenon may rely on primary tumor resistance or on the rapid onset of acquired escape mechanisms.

The identification of biomarkers of response and resistance toward AKT inhibitors is crucial, but currently represents a conundrum. Indeed, the results observed in a positive biomarker-selected population which should have benefited from AKT inhibition, are inconsistent and sometimes counterintuitive. Overall, the influence of PI3K/AKT alterations on AKT inhibitors seems to be strongly dependent on the specific biological context. In fact, in the HR + setting, the benefit of AKT inhibitors is unrelated to PI3K/AKT/PTEN status, whereas pathway alterations probably have a role in TNBC. Future translational and correlative research might shed light on these complex and highly controversial issues.

Inhibition of the PI3K pathway *in vivo* determines a well-known toxicity spectrum (hyperglycemia, skin rash, diarrhea, and mucositis) that is often difficult to manage (Chia et al., 2015; Esposito et al., 2019). Safety data from AKT inhibitors trials are in line with what was previously observed with mTOR or PI3K inhibitors. Clinical trials and real-world experience suggest that a proactive attitude toward these adverse events is key to their proper management, in order to preserve patient quality of life and minimize treatment delays or interruptions (Nunnery and Mayer, 2019).

While confirmatory phase III trials of AKT inhibitors in association with conventional therapy are still ongoing, new strategies to boost the efficacy of these compounds are already under way. A biological rationale exists to combine AKT inhibitors with CDK4/6 inhibitors, immunotherapeutic agents or PARP-inhibitors and the results from these studies are eagerly awaited. However, the higher the number of combined compounds, the higher the risk of additional or cumulative toxicities. Hence, an actual concern for the development of multiple combination regimens incorporating AKT inhibitors may be represented by their safety profile.

Results from ongoing trials will establish whether AKT inhibitors will join the therapeutic armamentarium available for the fight against breast cancer. Meanwhile, a concerted effort is needed to identify biomarkers of response, to reduce the toxicity burden of these compounds and to develop novel and effective combinations strategies, in order to complete the itinerary of AKT inhibitors from bench to clinical practice (Tirrò et al., 2019).
